# Supplementary material for: Optical Spike Detection and Connectivity Analysis With a Far-Red Voltage-Sensitive Fluorophore Reveals Changes to Network Connectivity in Development and Disease
Source: Front Neurosci. 2021 May 13;15:643859. doi: 10.3389/fnins.2021.643859 (PMC8155641; doi:10.3389/fnins.2021.643859)
Supplement: Supplementary Figure 1 — Optimization of BeRST 1 light power. Representative raw fluorescence traces normalized to baseline fluorescence values during a 30 s imaging bout used to score neurons as (a) healthy or (b) unhealthy. Neurons scored as (a) healthy have just a single, linear baseline, while neurons scored as (b) unhealthy have a change in the slope of the baseline (upper and middle trace), non-linear rises in baseline (middle trace), or abrupt steps in the baseline (lower trace). (c) Plot of the fluorescence intensity of the baseline for neurons characterized as either healthy or unhealthy at 500 nM or 1 μM BeRST 1 dye loading. Each point represents the baseline fluorescence intensity of a single neuron. Bars plot shows the mean fluorescence intensity and error bars are ± standard error of the mean. (d) Plot of fraction of healthy neurons (%) vs. illumination intensity. At lower BeRST 1 concentrations (500 nM, blue), higher light intensities (up to 31 mW/mm2) could be used without any signs of unhealthy neurons (as defined above). At higher concentrations of BeRST 1 (1 μM, red), lower light intensities had to be used (<17 mW/mm2) to avoid unhealthy neurons. Numbers next to data points are the number of neurons analyzed. (e) Plot of SNR per AP vs. relative fluorescence intensity for BeRST 1 loading at 500 nM (circles) and 1 μM (triangles), using between 6.5 and 70 mW/mm2 excitation power (same excitation light power scale as in “c”). [file Data_Sheet_1.PDF]

1.1 Supplementary Figures

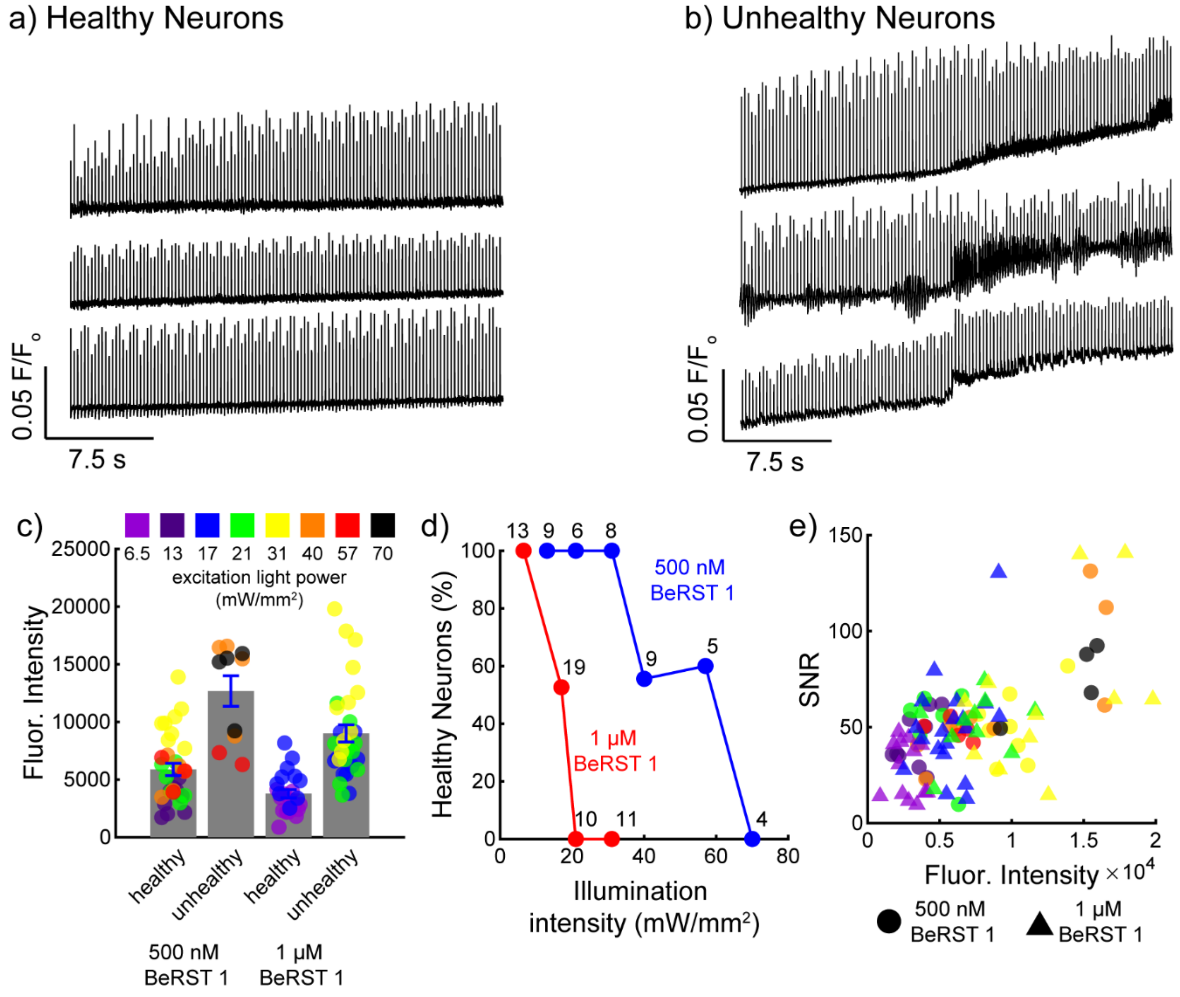

1.2 **Figure S1. Optimization of BeRST 1 light power.**

Representative raw fluorescence traces normalized to baseline fluorescence values during a 30 second imaging bout used to score neurons as **a**) healthy or **b**) unhealthy. Neurons scored as **a)** healthy have just a single, linear baseline, while neurons scored as **b)** unhealthy have a change in the slope of the baseline (upper and middle trace), non-linear rises in baseline (middle trace), or abrupt steps in the baseline (lower trace). **c**) Plot of the fluorescence intensity of the baseline for neurons characterized as either healthy or unhealthy at 500 nM or 1 µM BeRST 1 dye loading. Each point represents the baseline fluorescence intensity of a single neuron. Bars plot shows the mean fluorescence intensity and error bars are ± standard error of the mean. **d)** Plot of fraction of healthy neurons (%) vs. illumination intensity. At lower BeRST 1 concentrations (500 nM, blue), higher light intensities (up to 31 mW/mm^2) could be used without any signs of unhealthy neurons (as defined above). At higher concentrations of BeRST 1 (1 µM, red), lower light intensities had to be used (<17 mW/mm^2) to avoid unhealthy neurons. **e)** Plot of SNR per AP vs. relative fluorescence intensity for BeRST 1 loading at 500 nM (circles) and 1 µM (triangles), using between 6.5 and 70 mW/mm^2 excitation power (same excitation light power scale as in “c”).
